# Species prevalence, virulence genes, and antibiotic resistance of enterococci from food-producing animals at a slaughterhouse in Turkey

**DOI:** 10.1038/s41598-024-63984-y

**Published:** 2024-06-08

**Authors:** Tugba Cebeci

**Affiliations:** Department of Medical Services and Techniques, Espiye Vocational School, Giresun University, Giresun, Turkey

**Keywords:** Antibiotic resistance, Food-producing animal carcass, *Enterococcus* spp., MALDI-TOF MS, Slaughterhouse, Antimicrobials, Bacteriology, Microbial genetics, Microbiology, Public health

## Abstract

Healthy cattle, sheep, and goats can be reservoirs for gastrointestinal pathogenic fecal enterococci, some of which could be multidrug-resistant to antimicrobials. The objective of this study was to determine the prevalence and diversity of *Enterococcus* species in healthy sheep, goat, and cattle carcasses, as well as to analyze the antimicrobial resistance phenotype/genotype and the virulence gene content. During 2019–2020, carcass surface samples were collected from 150 ruminants in a slaughterhouse. A total of 90 enterococci, comprising five species, were obtained. The overall prevalence of enterococci was found to be 60%, out of which 37.7% were identified as *Enterococcus* (*E.*) *hirae*, 33.3% as *E. casseliflavus*, 15.5% as *E. faecium*, 12.2% as *E. faecalis*, and 1.1% as *E. gallinarum*. Virulence-associated genes of *efaA* (12.2%) were commonly observed in the *Enterococcus* isolates, followed by *gelE* (3.3%), *asaI* (3.3%), and *ace* (2.2%). High resistance to quinupristin-dalfopristin (28.8%), tetracycline (21.1%), ampicillin (20%), and rifampin (15.5%) was found in two, four, four, and five of the *Enterococcus* species group, respectively. The resistance of *Enterococcus* isolates to 11 antibiotic groups was determined and multidrug resistant (MDR) strains were found in 18.8% of *Enterococcus* isolates. Characteristic resistance genes were identified by PCR with an incidence of 6.6%, 2.2%, 1.1%, 1.1%, 1.1%, and 1.1% for the *tetM, ermB, ermA, aac(6ʹ)Ie-aph(2")-la, VanC1, and VanC2* genes in *Enterococcus* isolates, respectively. Efflux pump genes causing multidrug resistance were detected in *Enterococcus* isolates (34.4%). The results showed that there were enterococci in the slaughterhouse with a number of genes linked to virulence that could be harmful to human health.

## Introduction

Foodborne illnesses frequently occur after consuming contaminated food, particularly animal-derived products like meat^1^. Enterococci of animal origin are found in animal-derived foods that are consumed by humans^2^. Enterococci are a component of the natural microbiota in the digestive systems of animals and humans, particularly *Enterococcus faecalis* and *Enterococcus faecium,* which have been shown to be significantly important. These nosocomial pathogens are recognized as the causative agents of various animal ailments, including mastitis, endocarditis, diarrhea, and septicemia in cattle, domesticated animals, swine, and poultry^3–5^. The species *Enterococcus durans*, *Enterococcus hirae*, *Enterococcus gallinarum*, *Enterococcus cassseliflavus*, *Enterococcus faecium*, and *Enterococcus faecalis* are frequently present in the gastrointestinal systems of livestock^6^.

Enterococci’s pathogenesis is linked to a diverse range of virulence factors. Virulence factors contribute to the development of enterococcal infections by facilitating the attachment, colonization, and invasion of host tissues. They also affect the host’s immune response and produce enzymes and toxins outside of cells, which worsen the severity of the illness. The key adhesion factors involved in biofilm development include *Ebp* (endocarditis and biofilm-associated pili), *Asa* (aggregation substance), *EfaA* (*E. faecalis* antigen A), *Esp* (extracellular surface protein), *Ace* (collagen-binding cell wall protein), *cylA* (hemolysin), *efm* (*E. faecium*-specific cell wall adhesin), *cad1* (pheromone cAD1 precursor lipoprotein), *sagA* (secreted antigen), and *cpd1* (pheromone cPD1 lipoprotein)^7–9^.

The excessive use of antibiotics in animals is associated with the emergence of antimicrobial resistance, and mechanisms of antibiotic resistance can readily disseminate among all pathogenic, commensal, and environmental bacteria through horizontal gene transfer of mobile genetic elements^10,11^. Both human and animal enterococci possess intrinsic resistance to several antimicrobial drugs, and they also have the ability to develop resistance to additional antimicrobial agents, such as glycopeptides, quinolones, tetracyclines, macrolides, and streptogramins^1,12,13^. Although food-producing animals may not always directly transmit enterococci to humans, they can nonetheless facilitate the transfer of resistance genes from these animals to humans. Hence, the occurrence of resistant enterococci, particularly vancomycin-resistant enterococci, in animals used for food production has emerged as a significant issue^12^.

The emergence of antimicrobial resistance in zoonotic bacteria poses a substantial risk to public health, mainly owing to the heightened likelihood of treatment failures. Furthermore, the emergence of resistance, particularly through the acquisition of transmissible genetic components, might also impact other characteristics, such as the capacity to inhabit an animal host or endure in agricultural or food-processing settings^14,15^. The presence of antibiotic-resistant enterococci in meat, animal-related sources, and habitats linked to animals, food-handling equipment, and healthy humans emphasizes the importance of evaluating enterococci in slaughterhouses as well. The main source of zoonotic pathogens is the GI tract of healthy food animals. Most food-related diseases are spread by feces during the slaughtering process or by cross-contamination during processing^14,16^. The transfer of harmful microorganisms from one part of an animal’s body to another during the slaughtering process poses a substantial risk to the safety of the meat. Carcass tissues primarily become contaminated with fecal particles during the evisceration and skinning processes^17–19^. The aim of this study was to assess and characterize the prevalence, types, virulence determinants, and antimicrobial resistance profiles of enterococci from healthy cattle, sheep, and goat carcasses to highlight their zoonotic importance.

## Materials and methods

### Sample collection

Between November 2019 and December 2020, the researchers obtained carcass samples from a total of 20 cattle, 80 sheep, and 50 goats from a slaughterhouse located in Van, a city in the eastern region of Turkey. The animals that were sampled were chosen in a randomized way. Sampling was completed by visiting the slaughterhouse twice a year, depending on its slaughtering capacity. The selected slaughterhouse was visited at two occasions to obtain 600 surface swab samples from 150 animals (visit I, 10 cattle, 40 sheep, and 25 goats; visit II, 10 cattle, 40 sheep, and 25 goats) and carcasses during the pre-chilling stage of the slaughtering process. A total of 600 samples from different parts of beef, sheep, and goat carcasses were collected using swabs. The sampling locations were situated in the brisket, flank, hind leg, and rectal regions of the carcass. The sampling region was meticulously surveyed for 1 min using cotton swabs that were swiped in both vertical and horizontal directions. Four 100 cm^2^ areas, measuring 10 cm^2^ × 10 cm^2^, were swabbed on each beef, sheep, and goat carcass. Carcass swabs were collected pre-chilling using sterile cotton swabs soaked in 10 ml of buffered peptone water, following the protocols set by the International Organization for Standardization^20^. The sampler was pressed firmly and evenly as it was inserted vertically onto the peripheral surfaces, with this process repeated approximately 10 times. Then, the sampler was turned and used to swipe horizontally and diagonally, each motion being repeated around 10 times. The samples were promptly delivered to the laboratory of Espiye Vocational School, Giresun University, within 24–48 h of being collected, using refrigerated containers^21^.

### *Enterococcus* species isolation

Swab samples were homogenized in a blender (Waring, New Hartford, Conn.) with 90 ml of buffered peptone water (BPW) (Lab M, Lancashire, UK). After incubation at 37 °C for 24 h, 0.1 ml was streaked onto Slanetz and Bartley Agar (Lab M, Lancashire, UK) and incubated for 24 ± 2 h at 37 ± 1 °C under the same condition^3^. After incubation period, pink or dark red colonies with a narrow, whitish border were observed. After the incubation period, five colonies that showed characteristics of *Enterococcus* species were selected from each petri dish and transferred to Tryptone Soya Agar (Lab M, Lancashire, UK) agar for purification. The agar plates were then incubated at a temperature of 37 ± 1 °C for 24 ± 2 h. The suspected isolates were biochemically identified using Gram staining and catalase activity. All strains were stored in skimmed milk powder stocks at − 80 °C for further testing^22^. The *Enterococcus* species were identified through MALDI-TOF MS (BioMérieux Inc., Marcy l’Etoile, France), which was performed only on Gram-positive and catalase-negative cocci^23^.

### DNA isolation protocols

The QIAsymphony, a magnetic particle-based automated extraction system, was used to extract genomic DNA. The extraction was carried out using the QIAamp DNA micro kit (Qiagen, Hilden, Germany) following the instructions provided by the manufacturer. The isolated DNA was utilized as a template for PCR using the specified methods.

### Screening for confirmation and virulence genes

All *Enterococcus* isolates were screened for the confirmation genes and the presence of virulence genes. These were 16S rDNA^24^, *E. faecalis*^25^, *E. faecium*^25^, *E. casseliflavus*^25^, *E. gallinarum*^25^, *E. hirae*^26^ identification genes and virulence genes; *asa1* (aggregation substance)^27^, *ace* (collagen-binding protein)^28^, *cylA* (cytolysin activator)^27^, *efaA* (endocarditis antigen)^29^, *esp* (enterococcal surface protein)^28^, *gelE* (gelatinase)^28^, *hyl* (hyaluronidase)^28^. The methods reported Jahan et al.^24^ were modified and used for genotyping the *Enterococcus* isolates.

### Antimicrobial susceptibility testing

The susceptibility of *Enterococcus* isolates to antibiotics was assessed using the disc diffusion method, following the protocols outlined by the Clinical and Laboratory Standards Institute^30^. To determine antibiotic resistance in the isolates, 10 µg ampicillin (AM), 5 µg ciprofloxacin (CIP), 30 μg chloramphenicol (C), 15 µg erythromycin (E), 200 µg fosfomycin (FF), 300 μg high-level streptomycin-resistant (HLSR), 120 μg high-level gentamicin-resistant (HLGR), 10 μg imipenem (IPM), 5 µg levofloxacin (LEV), 30 μg linezolid (LNZ), 300 µg nitrofurantoin (F), 10 units penicillin (P), 15 μg quinupristin-dalfopristin (QD), 5 µg rifampin (RA), 30 µg vancomycin (VA), 30 µg teicoplanin (TEC), 30 µg tetracycline (TE), 15 μg tigecycline (TIG) and 5 μg vancomycin (VA) antibiotic discs were used (all purchased from Liofilchem, Roseto degli Abruzzi, Italy). After incubation, the resulting diameters of the inhibition zones that formed around the discs of AM, P, VA-30 µg, TEC, E, TE, CIP, LEV, F, RA, FF, C, QD, LNZ, HLSR, and HLGR were classified as susceptible, intermediate, or resistant according to the diameters and breakpoints reported in CLSI documents^30^. For the remaining antimicrobial agents (TIG, VA-5 µg, and IPM), the critical values were evaluated according to the zone table provided by the European Committee on Antimicrobial Susceptibility Testing document^31^. For quality control purposes, *Staphylococcus aureus* ATCC 25,923 and *E. faecalis* ATCC 29,212 were utilized as control strains.

### PCR detection of genes for antimicrobial resistance

In the present study, various PCR assays were used for the detection of antibiotic resistance genes (AGRs) in *Enterococcus* isolates. All isolates were tested for the presence of aminoglycoside modifying enzyme (AME) genes [*aac*(6*ʹ*)-*Ie*–*aph*(2ʺ)-*Ia*, *aph*(2ʺ)-*Ib*, *aph*(2ʺ)-*Ic*, *aph*(2ʺ)-*Id*, *ant*(3ʺ)-*Ia*, and *ant*(6)-*Ia*], phenicol resistance genes (*cfr*, *fexA*, and *optrA*), tetracycline resistance genes [*tet*(L), *tet*(M), and *tet*(O)], macrolide resistance genes [*ermA*, *ermB*, and *mef*], and efflux pump genes [*efr*(A), *emeA*, and *lsa*] by PCR using specific primers as described by a previous study^32^, with some modifications.

### Statistical analysis

Descriptive statistics for the categorical variables in this study, namely animal species, animal carcass sites, and *Enterococcus* species are expressed as a number (n) and a percentage (%). Chi-square and Fisher’s exact tests were used to determine the relationships between the factors animal species, carcass sites, and *Enterococcus* species. The SPSS (IBM SPSS for Windows, ver. 26) statistical package program was used for the analyses and the statistical significance level was set to *p* < 0.05.

### Ethical approval and consent to participate

Ethical review and approval were waived for this study, since all sample materials were carcass surface swab samples and were collected during authorized slaughtering in registered slaughterhouses for human consumption. No animals were slaughtered for research purposes.

## Results

### Prevalence of enterococci

The overall prevalence of enterococci in sheep, goat, and cattle animals from a slaughterhouse in Van, Turkey was 60% (90/150). The species distribution is shown in Table 1. The predominant species detected were *E. hirae* (*n* = 34, 37.7%) and *E. casseliflavus* (*n* = 30, 33.3%). A smaller number of *E. faecium* (*n* = 14, 15.5%), *E. faecalis* (*n* = 11, 12.2%), and *E. gallinarum* (*n* = 1, 1.1%) were also evaluated. The number of *Enterococcus* species in the brisket, flank, hind leg, and rectal samples of carcasses is shown in Table 1. *Enterococcus* species contamination was not significantly different in animal species in comparison to carcass sites (*p* < 0.05). *E. casseliflavus* and *E. faecium* were isolated from 10 and 6 out of 150 carcass surface samples from the brisket and hind leg, respectively, whereas *E. hirae*, *E. faecalis*, and *E. gallinarum* were isolated from 13, 5, and 1 out of 150 carcass surface samples from rectal swabs, respectively.

**Table 1:** Prevalence of *Enterococci* in brisket, flank, hind leg, and rectal swab samples.

Animal species	Carcass surface point	Sample number	*E. faecalis*	*E. hirae*	*E. faecium*	*E. casseliflavus*	*E. gallinarum*	Total
			n (%)	n (%)	n (%)	n (%)	n (%)	n (%)
Sheep	Brisket	80	0 (0)	4 (11.7)	0 (0)	7 (23.3)	0 (0)	11 (12.2)
	Flank	80	1 (9.09)	5 (14.7)	2 (14.2)	1 (3.3)	0 (0)	9 (10)
	Hind leg	80	2 (18.1)	4 (11.7)	6 (42.8)	4 (13.3)	0 (0)	16 (17.7)
	Rectal	80	4 (36.3)	8 (23.5)	2 (14.2)	4 (13.3)	0 (0)	18 (20)
Goat	Brisket	50	1 (9.09)	1 (2.9)	0 (0)	3 (10)	0 (0)	5 (5.5)
	Flank	50	0 (0)	1 (2.9)	1 (7.1)	2 (6.6)	0 (0)	4 (4.4)
	Hind leg	50	0 (0)	6 (17.6)	0 (0)	4 (13.3)	0 (0)	10 (11.1)
	Rectal	50	1 (9.09)	5 (14.7)	3 (21.4)	5 (16.6)	0 (0)	14 (15.5)
Cattle	Brisket	20	0 (0)	0 (0)	0 (0)	0 (0)	0 (0)	0 (0)
	Flank	20	2 (18.1)	0 (0)	0 (0)	0 (0)	0 (0)	2 (2.2)
	Hind leg	20	0 (0)	0 (0)	0 (0)	0 (0)	0 (0)	0 (0)
	Rectal	20	0 (0)	0 (0)	0 (0)	0 (0)	1 (100)	1 (1.1)
	Total	600	11 (12.2)	34 (37.7)	14 (15.5)	30 (33.3)	1 (1.1)	90

Enterococci were detected in a total of 150 animal species, comprising 60% sheep, 36.6% goats, and 3.3% cattle. There was significant difference in prevalence (*p* < 0.010) for the different *Enterococcus* species across different animal species.

### Virulence of Enterococci

The distribution of virulence genes among *Enterococcus* species is presented in Table 2. The different species of *Enterococcus* showed variability in their virulence gene profiles. The hyaluronidase virulence factor *hyl*, enterococcal surface protein *esp,* and cytolysin activator gene *cylA* were absent in all 90 of the *Enterococcus* isolates*.* Among the *E. faecalis* isolates, six (54.5%) isolates tested positive for the *efaA* gene*. The* aggregation substance gene, *asaI,* was found in two (18.8%) *E. faecalis* isolates. The collagen-binding protein gene, *ace,* and the gelatinase gene, *gelE,* were possessed by one (9.9%) and one (9.9%) *E. faecalis* isolates, respectively. Two, two, and one of five *E. hirae* isolates tested positive for *gelE*, *efaA,* and *asaI* genes, respectively, whereas none of them possessed the *ace, esp, cylA,* or *hyl* genes. For *E. faecium* and *E. casseliflavus* isolates, 3/14 (21.4%) and 1/30 (3.3%) harbored *efaA* and *ace* genes, respectively. Table 3 displays the incidence of virulence genes in enterococci from sheep, goats, and cattle. The genes *ace*, *gelE*, *efaA,* and *asaI* were isolated from sheep species, whereas *efaA* and *asaI* were isolated from goat species.

**Table 2:** Distribution of virulence genes profiles among enterococci.

Virulence genes	Number (%) of *Enterococcus* virulence factor genotypes					
	*E. faecalis* n = 11	*E. hirae* n = 34	*E. faecium* n = 14	*E.casseliflavus* n = 30	*E.gallinarum* n = 1	Total (n = 90)
	n (%)	n (%)	n (%)	n (%)	n (%)	n (%)
*ace*	1 (9.09)	0 (0)	0 (0)	1 (3.3)	0 (0)	2 (2.2)
*gelE*	1 (9.09)	2 (18.8)	0 (0)	0 (0)	0 (0)	3 (3.3)
*efaA*	6 (54.5)	2 (18.8)	3 (21.4)	0 (0)	0 (0)	11 (12.2)
*esp*	0 (0)	0 (0)	0 (0)	0 (0)	0 (0)	0 (0)
*asaI*	2 (18.8)	1 (2.9)	0 (0)	0 (0)	0 (0)	3 (3.3)
*cylA*	0 (0)	0 (0)	0 (0)	0 (0)	0 (0)	0 (0)
*hyl*	0 (0)	0 (0)	0 (0)	0 (0)	0 (0)	0 (0)

**Table 3:** Distribution of virulence genes in enterococci from sheep, goat and cattle.

Virulence genes	Animal species			
	Cattle n = 20	Sheep n = 80	Goat n = 50	Total (n = 150)
	n (%)	n (%)	n (%)	n (%)
*ace*	0 (0)	2 82.5)	0 (0)	2 (1.3)
*gelE*	0 (0)	3 (3.75)	0 (0)	3 (2)
*efaA*	0 (0)	7 (8.7)	4 (8)	11 (7.3)
*esp*	0 (0)	0 (0)	0 (0)	0 (0)
*asaI*	0 (0)	1 (1.2)	2 (4)	3 (2)
*cylA*	0 (0)	0 (0)	0 (0)	0 (0)
*hyl*	0 (0)	0 (0)	0 (0)	0 (0)

### Antibiotic resistance

The resistance patterns of enterococci towards the tested antimicrobial agents are presented in Table 4. Resistance to QD was the most common (28.8%), followed by TE (21.1%), AM (18%), and RA (15.5%). High rates of resistance to QD were found in *E. hirae* (21.1%) and *E. faecium* (7.7%). Tetracycline resistance was common in *E. faecalis* (6.6%), *E. casseliflavus* (6.6%), *E. hirae* (4.4%), and *E. faecium* (3.3%). Ampicillin and rifampin resistance were frequent in *E. faecium* (8.8% and 4.4%, respectively). Resistance to VA (7.7%), CIP (6.6%), F (4.4%), FF (4.4%), C (4.4%), P (2.2%), LEV (1.1%), and TIG (1.1%) was relatively low. HLSR (5.5%) resistance was found in a few of the isolates from *E. faecalis, E. faecium,* and *E. casseliflavus,* but not in any from *E. gallinarum*. A total of 90 isolates from four tested species were resistant to at least one antibiotic (64.4%, 58/90), with 17 isolates (18.8%, 17/90) from 90 *Enterococcus* isolates displaying multidrug resistance (Tables 4 and 5).

**Table 4:** Antimicrobial resistance pattern of *Enterococus* species.

Antibiotic group	Antibiotics	*Enterococcus* species					
		*E. faecalis* n = 11	*E. hirae* n = 34	*E. faecium* n = 14	*E.casseliflavus* n = 30	*E. gallinarum* n = 1	Total n = 90
		n (%)	n (%)	n (%)	n (%)	n (%)	n (%)
Penicillins	AM	–	5 (5.5)	8 (8.8)	4 (4.4)	1 (1.1)	18 (20)
Penicillins	P	–	–	2 (2.2)	–	–	2 (2.2)
Lipoglycopeptides	TEC	–	–	–	–	–	–
Macrolides	E	*	3 (3.3)	*	*	*	3 (3.3)
Tetracyclines	TE	6 (6.6)	4 (4.4)	3 (3.3)	6 (6.6)	–	19 (21.1)
Fluoroquinolones	CIP	1 (1.1)	–	5 (5.5)	–	–	6 (6.6)
Fluoroquinolones	LEV	1 (1.1)	–	-	–	–	1 (1.1)
Nitrofurans	F	–	4 (4.4)	-	–	–	4 (4.4)
Ansamycins	RA	2 (2.2)	4 (4.4)	4 (4.4)	3 (3.3)	1 (1.1)	14 (15.5)
Fosfomycins	FF	–	–	2 (2.2)	2 (2.2)	–	4 (4.4)
Phenicols	C	2 (2.2)	–	1 (1.1)	1 (1.1)	–	4 (4.4)
Streptogramins	QD	*	19 (21.1)	7 (7.7)	*	*	26 (28.8)
Oxazolidinones	LNZ	–	–	–	–	–	–
Tetracyclines	TIG	–	–	–	1 (1.1)	–	1 (1.1)
Carbapenems	IPM	–	–	–	–	–	–
Glycopeptides	VA	3 (3.3)	3 (3.3)	1 (1.1)	–	–	7 (7.7)
Aminoglycosides	HLSR	1 (1.1)	–	2 (2.2)	2 (2.2)	–	5 (5.5)
Aminoglycosides	HLGR	–	–	–	–	–	–
MDR		4 (36.3)	4 (11.7)	7 (30)	2 (6.6)		17 (18.8)

*Intrinsic resistance; *AM* ampicillin; *P* penicillin; *VA* vancomycin; *TEC* teicoplanin; *E* erythromycin; *TE* tetracycline; *CIP* ciprofloxacin; *LEV* levofloxacin; *F* nitrofurantoin; *RA* rifampin; *FF* fosfomycin; *C* chloramphenicol; *QD* quinupristin-dalfopristin; *LNZ* linezolid; *TIG* tigecycline; *IPM* imipenem; *HLSR* high-level streptomycin-resistant; *HLGR* high-level gentamicin-resistant; *VA* vancomycin; *MDR* multidrug resistance.

**Table 5:** Characteristics of the 17 multidrug-resistant strains of enterococci.

Strain	Animal species	Carcass part	Antibiotic resistance		Virulence factor
			Phenotype	Genotype	
EFM-4	Sheep	Hind leg	CIP, RA, QD		
EFM-7	Sheep	Hind leg	RA,P,AM		
EC-39	Goat	Rectal	HLSR, TE, AM		
EFM-45	Sheep	Rectal	CIP, RA, TE		
EH-48	Sheep	Rectal	E, AM, QD		
EH-49	Sheep	Brisket	RA, AM, QD	*ermB*	
EFM-57	Sheep	Rectal	HLSR, FF, AM,		
EH-66	Goat	Hind leg	RA, E, QD		
EFS-76	Sheep	Hind leg	C, CIP, LEV, RA, TE, AM	*aac(6’)Ie-aph(2’’)-la*, *Isa*, *efrA*, *tetM*	*efaA*
EFM-88	Goat	Flank	VA, AM, QD	*VanC1*	
EFS-97	Goat	Rectal	VA, C, HLSR, TE	*Isa*, *efrA*, *emeA*, *tetM*	*efaA*
EC-98	Sheep	Brisket	C, HLSR, AM, TE		
EFM-99	Goat	Rectal	C, HLSR, TE, FF, QD	*Isa*, *efrA*, *tetM*	
EFS-106	Cattle	Brisket	VA, TE, AM	*Isa, tetM*	
EFS-108	Sheep	Flank	VA, RA, TE, AM, QD	*Isa, tetM*	
EFM-113	Sheep	Hind leg	CIP, RA, TE, P, AM		
EH-116	Goat	Hind leg	F, E, AM		

EFM, *Enterococcus faecium*; EH, *Enterococcus hirae*; EFS, *Enterococcus faecalis*; EC, *Enterococcus casseliflavus*; *AM* ampicillin; *P* penicillin; *E* erythromycin; *TE* tetracycline; *CIP* ciprofloxacin; *LEV* levofloxacin; *F* nitrofurantoin; *RA* rifampin; *FF* fosfomycin; *C* chloramphenicol; *QD* quinupristin-dalfopristin; *HLSR* high-level streptomycin-resistant.

### Genotyping of antibiotic resistance

The distribution of antibiotic resistance genes amongst *Enterococcus* species is presented in Table 6 and Fig. S1. Of the three isolates showing resistance to E, one (1.1%) carried the *ermA* gene, and two (2.2%) carried *aac(6’)Ie-aph(2")-la*. Three efflux pump genes, *efr*(A), *emeA*, and *lsa*, were observed in 31 *Enterococcus* isolates. Glycopeptide genes *VanC1* and *VanC2* were present in 1.1% and 1.1% of *E. faecium* and *E. hirae* isolates in goat and sheep carcasses, respectively.

**Table 6:** The presence of antibiotic resistance genes profiles among enterococci.

Antibiotic resistance genes	*Enterococcus* species					
	*E. faecalis* n = 11	*E. hirae* n = 34	*E. faecium* n = 14	*E.casseliflavus* n = 30	*E. gallinarum* n = 1	Total (n = 90)
	n (%)	n (%)	n (%)	n (%)	n (%)	n (%)
*ermA*	*	1 (1.1)	*	*	*	1 (1.1)
*ermB*	*	2 (2.2)	*	*	*	2 (2.2)
*mef*	*	–	*	*	*	–
*tet(L)*	–	–	–	–	–	–
*tet(M)*	5 (5.5)	–	–	1 (1.1)	–	6 (6.6)
*tet(O)*	–	–	–	–	–	–
*cfr*	–	–	–	–	–	–
*fexA*	–	–	–	–	–	–
*optrA*	–	–	–	–	–	–
*aac(6’)Ie-aph(2")-la*	1 (1.1)	–	–	–	–	1 (1.1)
*aph(2")-Ib*	–	–	–	–	–	–
*aph(2")-Ic*	–	–	–	–	–	–
*aph(2")-Id*	–	–	–	–	–	–
*ant(3")-Ia*	–	–	–	–	–	–
*aph(6)-Ia*	–	–	–	–	–	–
*vanA*	–	–	–	*	*	–
*vanB*	–	–	–	*	*	–
*vanC1*	–	–	1 (1.1)	*	*	1 (1.1)
*vanC2*	–	1 (1.1)	–	*	*	1 (1.1)
*efr(A)*	5 (5.5)	2 (2.2)	1 (1.1)	1 (1.1)	–	9 (10)
*lsa*	9 (10)	2 (2.2)	1 (1.1)	1 (1.1)	–	13 (14.4)
*eme(A)*	6 (6.6)	1 (1.1)	–	2 (2.2)	–	9 (10)

*Intrinsic resistance, *n* number.

## Discussion

Enterococci, being a component of the normal microorganisms found in the GI of animals, can be present in meat during the slaughtering process. The prevalent species include *E. hirae, E. faecium*, *E. faecalis*, *E. casseliflavus*, *E. mundtii*, *E. durans*, and *E. gilvus*^33^. Transmission from other people, the environment, and foods contaminated with livestock intestinal microflora are just a few of the ways that enterococci can infect humans^34^. The objective of this study was to determine the frequency of *Enterococcus* species, analyze their patterns of antibiotic resistance, and identify the presence of resistance and virulence genes in the *Enterococcus* species collected from a slaughterhouse in Van, Turkey. This research is particularly relevant because of the high consumption of meat by a significant portion of the local population. Wide variation (0–90.6%) in the prevalence of enterococci in food-producing animals has been reported in this study and different countries^1,4,5,12,16,35–37^. In the present study, the speciation of the isolates confirmed that *E. hirae* was the most prevalent species identified from sheep and goat carcass samples. Other studies reported the prevalence of *Enterococcus* spp. on cattle in a slaughterhouse with a recovery rate of *E. hirae*, ranging from 8 to 92%^13,38^. Enterococci, especially *E. faecalis* and *E. faecium*, are known to be nosocomial pathogens and have become a major clinical concern^38^. Although *E. faecalis* and *E. faecium* isolates identified in this study have been found at low levels by Ramos et al.^1^, Holman et al.^36^ and Güngör et al.^39^ both species were isolated from slaughtered animals (12.2%, 15.5% and 7.2% respectively). *E. casseliflavus* and *E. gallinarum* were also isolated from sheep and cattle carcasses, supporting similar findings reported by Ramos et al.^1^ and Smoglica et al.^5^, respectively. The data regarding the occurrence of enterococci in cattle, sheep, and goat carcasses exhibits wide variation. Differences in the occurrence rates of enterococci in cattle, sheep, and goat carcasses may be attributed to variances in geographical regions, hygiene conditions, livestock management practices, agro-climatic factors, detection and sampling methods, animal breeds, and age. Potential factors influencing the variability in the results include the quality levels of the farms supplying the animals and the health and sickness conditions of the killed animals.

Given that enterococci are naturally found in the intestinal tract of animals, it is possible for meat to get contaminated during the slaughtering process. Various enterococcal virulence genes associated with the pathogenesis of disease in humans have been documented. It is essential to carry out genetic screening on enterococci to determine their capacity to cause disease and their ability to spread between from animals to humans, which poses a significant health risk. The genetic transmission mechanisms are closely interconnected with the virulence traits of particular enterococci^40–43^. The identification of virulence factors is crucial in assessing bacterial pathogenicity, as these factors enable microorganisms to invade and harm the host. In this study, virulence typing was conducted by targeting seven specific genes. The high prevalence (12.2%) of endocarditis antigen *efaA* in *E. faecalis*, *E. hirae,* and *E. faecium* was consistent with findings from previous reports, whereas the moderate presence of *ace*, *gelE*, and *asaI* was lower than that previously reported by Beukers et al.^10^, Zhang et al.^44^, and Güngör et al.^39^. Other authors have observed different values. Klibi et al.^12^ detected *gelE*, *especially* in 11.5% and 10% of *Enterococcus* isolates in fecal samples from animals in Tunisia, respectively. In Italy, Smoglica et al.^5^, observed the *gelE*, *asaI*, *efaA*, *ace,* and *esp* genes in 35.41%, 25%, 22.91%, 0.08%, and 0.04% of *Enterococcus* isolates, respectively. In another study, Song et al.^8^ reported that *E. faecalis* isolates were positive for *gelE* (88%), *asa1(44%), cylA* (16%), and *esp* (4%) virulence factor genes*.* The diversity in enterococci virulence genes reported from other studies might be attributable the different sampling techniques, sample types, isolation processes, environmental conditions, or geographic regions.

Enterococci are environmental organisms that have the ability to adapt to and spread antimicrobial-resistant traits^45^. Antimicrobial-resistant enterococci in animals are thought to serve as a reservoir for transmitting resistance genes to enterococci in humans. This transmission can occur through various means, such as human ingestion of animal-derived food, direct contact between animals and humans, or environmental factors. The preference of enterococci for certain hosts does not exclude the potential spread of antimicrobial resistance from animals to humans by enterococci^2^. In this investigation, *Enterococcus* isolates exhibited resistance to one or more antimicrobial agents, with a prevalence of 64.4%. The antibiotic resistance of *Enterococcus* isolates was assessed for 18 antimicrobial agents using the disk diffusion method. In this study, the high prevalence of tetracycline resistance, which was detected in enterococci other than antibiotics that belong to the natural resistance group, may be linked to the use of tetracyclines in the treatment of cattle, sheep, and goats. OTC is a tetracycline antibiotic primarily used to treat infections caused by a broad range of bacteria. However, it is important to note that resistance to OTC is frequently observed among Gram-negative bacilli of enteric origin and staphylococci^46,47^. One prevalent application is in the treatment of BRD in cattle, which is caused by *Pasteurella multocida*, *Mannheimia haemolytica*, and *Histophilus somni* (formerly known as *Haemophilus somnus*). Doxycycline is the preferred treatment for *Rickettsiae* and *Ehrlichiae* in small animals, as opposed to oxytetracycline^46,48^. Resistance to tetracycline in *Enterococcus* isolates was consistent with current results, which revealed a high resistance rate^1,4,49,50^, but a higher prevalence of tetracycline-resistant *E. faecalis* was obtained in the present study^51–53^. A study conducted in Tunisia between September 2011 and December 2011 showed that *E. hirae* species isolated from animal stools were resistant to tetracyclines, in accordance with the findings of this study^12^. The *E. faecium* isolates were more resistant to ampicillin antimicrobial agents (20%). This result was in accordance with another study in Saudi Arabia that revealed similar result^9^. Resistance to rifampin in *E. casseliflavus* was consistent with the current result, which revealed a low resistance rate^16^. Compared with other antimicrobial agents, the resistance rates to ciprofloxacin were relatively low in *E. faecium* and *E. faecalis* (1.1% and 2.2%, respectively). These findings produced parallel results to those of other studies conducted in the USA and Korea^8,36^. The prevalence of antimicrobial-resistant *Enterococcus* strains in slaughtering and production can be explained by the extensive utilization of antibiotics for growth promotion, disease prevention, and infection treatment.

Multidrug resistance in enterococci can arise from either intrinsic attributes of the species or from acquired resistance mechanisms. Resistance to aminoglycosides can be attributed to both intrinsic and acquired factors. Resistance to high concentrations of gentamicin and streptomycin is usually acquired through the transfer of resistant genes, while resistance to low concentrations of amikacin, tobramycin, and kanamycin is frequently caused by intrinsic factors^54,55^. In this study, we have observed HLSR and multidrug resitance in about 5.5% and 18.8% of *Enterococcus* isolates, whereas other studies have reported higher rates, including Li et al. (50%)^56^ and Ngbede et al. (53.1%)^49^, respectively. Research conducted on cattle in South Australia^16^. revealed that 26.9% of the isolates were resistant to at least two different classes of antibiotics. The values recorded by those authors were higher than the values noted in the current study (18.8%).

Enterococci can gain resistance to antibiotics through chromosomal mutations and horizontal gene transfer. Enterococci are naturally resistant to a wide range of antibiotic classes^15^. Thus, enterococci present a major challenge to illness treatment because of their limited susceptibility to antibiotics, which is caused by both intrinsic and acquired resistance. This resistance enables them to obtain supplementary resistances on mobile genetic elements, resulting in heightened interaction with other antibiotic-resistant microorganisms^57^. Overall, 22 target antibiotic-resistant and efflux pump genes were detected at variable frequencies in the tested isolates of *Enterococcus,* where *tet(M)*, *efr(A)*, *Isa,* and *eme(A)* were more commonly detected compared to the *ermA*, *ermB*, *vanC1, vanC2,* and *aac(6’)Ie-aph(2")-la* genes, which were detected at lower frequency (Table 6, Fig. S1). *E. faecalis* harbored a greater number of *tet(M)* gene (5 isolates) than the other *Enterococcus* isolates, in agreement the results reported from a previous study^9^. Holman et al.^36^ reported that the *tet(M)* antimicrobial resistance-encoding gene was detected in 31.9% (15 isolates) of *E. faecalis* bacteria. They also found that the *tet(M)* gene was detected in 37.5% (3 isolates) of *E. faecium*. Yu et al.^53^ reported similar results. They showed that the distribution of *tet(M) and* *aac(6’)Ie-aph(2")-la* resistance genes in *E. faecalis* strains isolated from poultry in China was 91.80% and 67.21%, respectively. A study carried out in Tunisian^12^ reported that *E. faecalis* isolated from food-producing animals carried *aac(6’)Ie-aph(2")* antimicrobial resistance-encoding genes. A total of 31 of the 85 *E. casseliflavus* isolates harbored the *tetM* gene. This result is similar to that reported for *E. casseliflavus* isolates from swine farms^58^. Out of the three genes *ermA*, *ermB*, and *mef*, *ermB* was the most frequently detected in this investigation. It was found in 5.8% of *E. hirae* isolates (n = 2/34), which is consistent with findings from other studies^1,10,12,41^. *VanC1* and *VanC2* were identified in *E. faecium* and *E. hirae* isolates, respectively. These results are consistent with a previous study that identified *VanC1/VanC2* resistance genes in *E. faecium* isolates from aquaculture and slaughterhouse facilities^41^. Efflux pumps play a significant role in both natural and acquired resistance to antimicrobial medicines that are currently employed for the treatment of infectious illnesses^59^. The finding of the present that over 34.4% of enterococcal isolates carried efflux pump genes is consistent with previous reports from bovine feces, retail chicken meat, and broiler chickens^10,32,60^. The changes in antibiotic use differs between nations because of the different variations use patterns. Efflux pumps and the acquisition of genetic elements such as plasmids and transposons can result in increased tolerance or resistance to antimicrobials in enterococci.

## Conclusions

The isolation of virulence potential and multidrug-resistant enterococci isolates from slaughtered cattle, sheep, and goat carcasses emphasized the importance of slaughter hygiene in the transmission of pathogenic enterococci. The presence of enterococci in different parts of the carcasses and during the pre-chilling stage poses a risk of cross-contamination in the examined facility. Carcasses can be contaminated with fecal bacteria, the majority of which results from contamination during the slaughtering process, such as damage to intestinal tissue during evisceration or fecal leakage, which can increase cross-contamination of carcasses. According to the the findings in this study, enterococci may pose a potential risk to public health, considering their virulence potential and antibiotic resistance (mainly against quinupristin-dalfopristin, tetracycline, and ampicillin). Therefore, considering the slaughterhouse in Van Province included in this study, larger studies from different geographical regions and more slaughterhouses or other sources of animals are needed to fully understand the genetic diversity of enterococci in farm animals.

### Supplementary Information

Supplementary Information.

## Data Availability

The data generated and analyzed in the current study are available from the corresponding author. All data generated or analyzed during this study are included in this article (and in the Supplementary Materials).

## Abbreviations

*E.*: Enterococcus
PCR: Polymerase chain reaction
MDR: Multidrug-resistant
MALDI-TOF MS: Matrix-assisted laser desorption/ionization time of flight mass spectrometry
GI tract: Gastrointestinal tract
ISO: International Organization for Standardization
CLSI: Clinical and Laboratory Standards Institute
EUCAST: European Committee on Antimicrobial Susceptibility Testing
AGRs: Antibiotic resistance genes
AME: Aminoglycoside modifying enzyme
OTC: Oxytetracycline
BRD: Respiratory disease
