# Supplementary material for: Species prevalence, virulence genes, and antibiotic resistance of enterococci from food-producing animals at a slaughterhouse in Turkey
Source: Sci Rep. 2024 Jun 8;14:13191. doi: 10.1038/s41598-024-63984-y (PMC11162463; doi:10.1038/s41598-024-63984-y)
Supplement: Supplementary file 1 — Supplementary Information. [file 41598_2024_63984_MOESM1_ESM.pdf]

Species Prevalence, Virulence Genes and Antibiotic Resistance of Enterococci from Food-producing Animals at a Slaughterhouse in Turkey

Tugba Cebeci[1*]

[1] Department of Medical Services and Techniques, Espiye Vocational School, Giresun University, Giresun,Turkey

Correspondence author Tugba Cebeci

Telephone number: +90 (454) 310 14 30 /6481

Fax number: +90 (454) 310 14 31

E-mail: tgbcbcdmn@gmail.com

ORCID-ID: 0000-0001-8960-0587

Supplementary Information for: Original version of the gel showing the presence of antibiotic resistance genes in enterococci

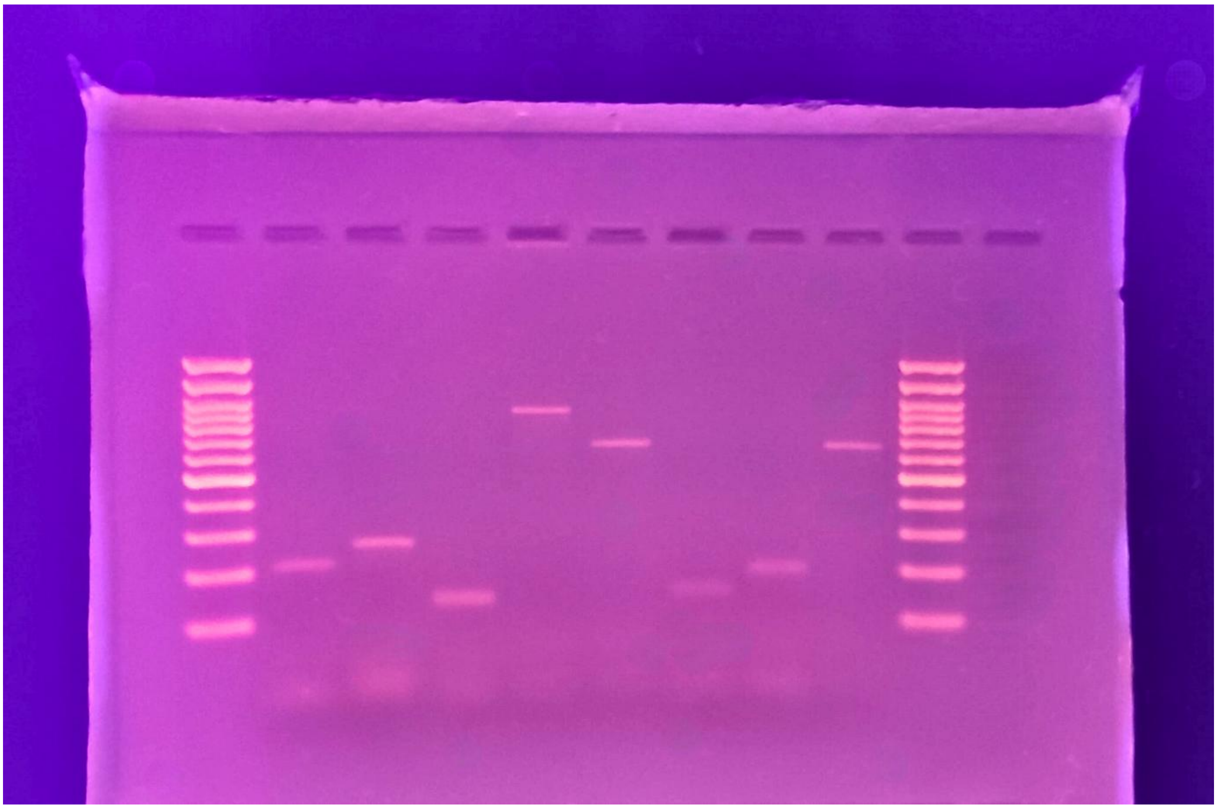

Figure 1. PCR assay revealing detection of genes for antibiotic resistance (*lsa*, *efr*(A), *emeA*, *vanC1*, *vanC2*, *ermA*, *ermB*, *tetM*) of *Enterococcus* isolates. lane M, DNA molecular weight marker (100 bp); Lane 1, *Enterococcus hirae lsa* 232 bp; lane 2, *Enterococcus faecalis efr(A)* 258 bp; lane 3, *Enterococcus casseliflavus emeA* 123 bp*; 4, Enterococcus faecium VanC1* 902 bp; lane 5, *Enterococcus hirae VanC2* 663 bp; lane 6, *Enterococcus hirae ermA* 200 bp; lane 7, *Enterococcus hirae ermB* 139 bp, lane 7; *Enterococcus faecalis tetM* 657 bp; lane M, DNA molecular weight marker (100 bp).
